# Supplementary figures and images for: Weak electric fields promote resonance in neuronal spiking activity: Analytical results from two-compartment cell and network models
Source: PLoS Comput Biol. 2019 Apr 22;15(4):e1006974. doi: 10.1371/journal.pcbi.1006974 (PMC6476479; doi:10.1371/journal.pcbi.1006974)

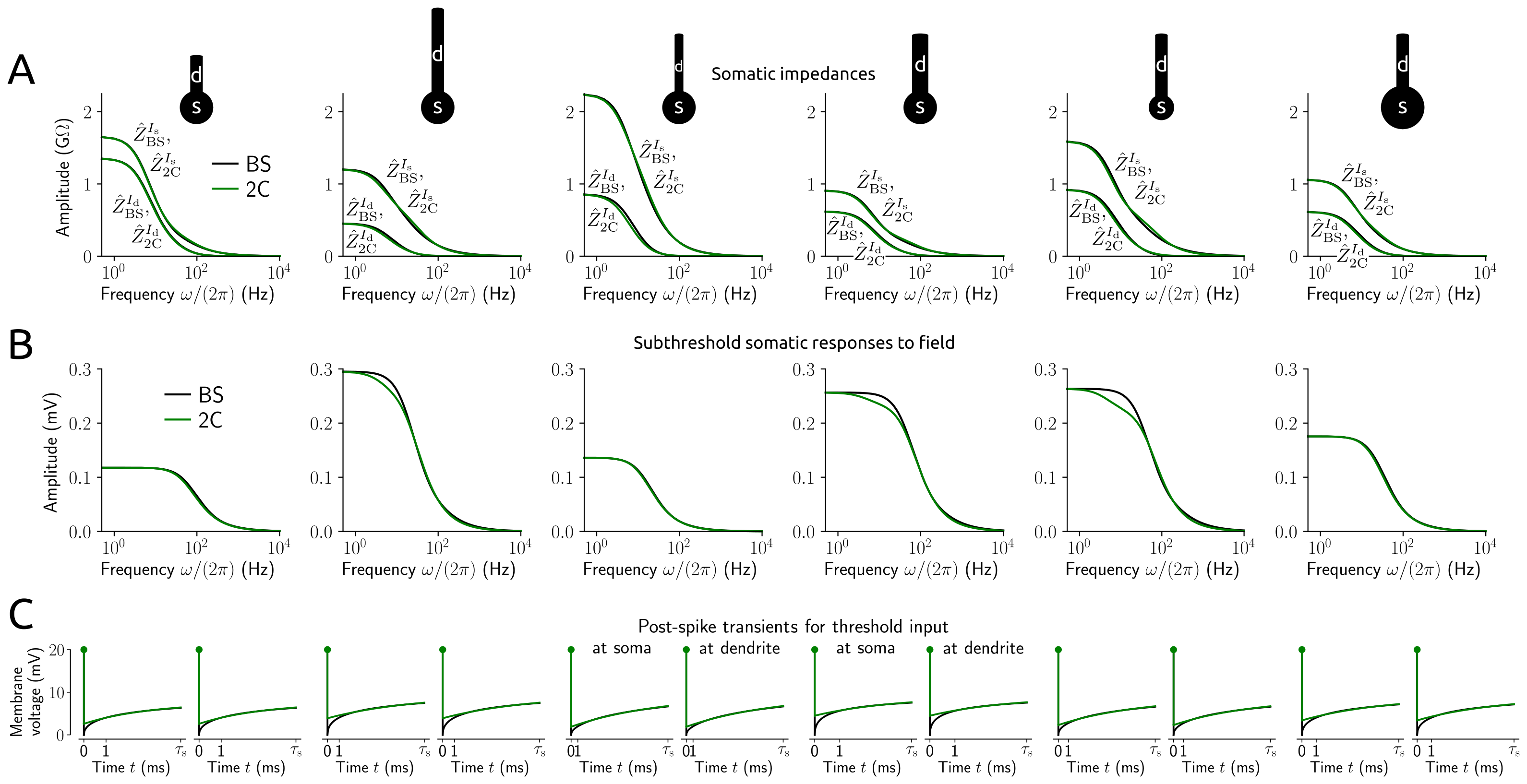

Supplement: S1 Fig — A: amplitude of subthreshold somatic impedances for inputs at the soma and dendrite, respectively, as a function of input frequency (cf. Fig 1C). B: amplitude of subthreshold somatic voltage responses to a sinusoidal electric field with amplitude E1 = 1 V/m as a function of field frequency (cf. Fig 1D). C: somatic voltage transients after a spike for constant threshold inputs at the soma and dendrite, respectively (cf. Fig 1E). The six columns correspond to different parametrizations (morphologies) of the ball-and-stick model, from left to right: L = 4 ⋅ 10−4 m, L = 10 ⋅ 10−4 m (default L = 7 ⋅ 10−4 m), Dd = 0.5 ⋅ 10−6 m, Dd = 1.5 ⋅ 10−6 m (default Dd = 1 ⋅ 10−6 m), Ds = 10 ⋅ 10−6 m, Ds = 20 ⋅ 10−6 m (default Ds = 15 ⋅ 10−6 m). For all other parameter values see Table 1. All curves were analytically computed. (TIFF) [file pcbi.1006974.s001.tiff]

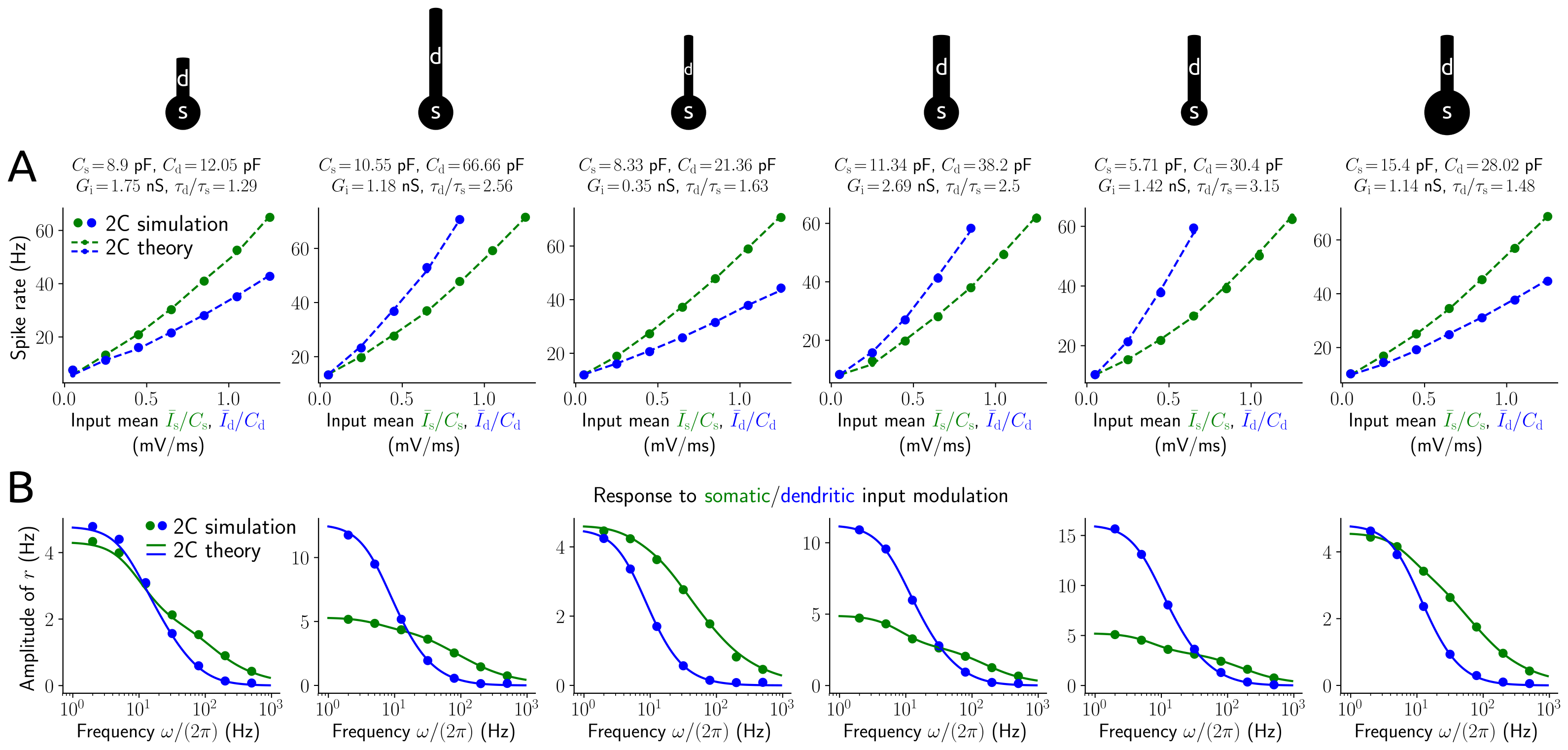

Supplement: S2 Fig — A: spike rate as a function of somatic (green) and dendritic (blue) mean input, respectively, for σs/Cs=0.1V/s and σd/Cd=0.05V/s (cf. Fig 1H). B: amplitude of spike rate responses to sinusoidal modulations of the mean input at the soma (green) or at the dendrite (blue) as a function of modulation frequency, for baseline input statistics I¯s0/Cs=I¯d0/Cd=0.25mV/ms, σs/Cs=0.1V/s and σd/Cd=0.05V/s (cf. Fig 2C and 2D). Modulation amplitudes were I¯s1/Cs=I¯d1/Cd=0.1mV/ms. Columns correspond to different parametrizations of the two-compartment model, obtained by fitting ball-and-stick neurons with different morphology (see S1 Fig). The values of critical parameters are indicated; somatic and dendritic time constants are defined by τs ≔ Cs/(Gs + Gi), τd ≔ Cd/(Gd + Gi). For comparison, the default (fitted) values were Cs = 9.9 pF, Cd = 28.9 pF, Gi = 1.2 nS, τd/τs = 2.04. Dots denote results from numerical simulations, solid or dashed curves from analytical calculations. Remark: spike rate responses to a weak sinusoidal field may be directly calculated from the responses to mean input modulations at the soma and dendrite, respectively, using both response amplitudes and phases (see Eq (57)). (TIFF) [file pcbi.1006974.s002.tiff]
